# Co-Occurrence of Gaming Disorder and Other Potentially Addictive Behaviours between Australia, New Zealand, and the United Kingdom

**DOI:** 10.3390/ijerph192316078

**Published:** 2022-12-01

**Authors:** Tyrone L. Burleigh, Mark D. Griffiths, Alexander Sumich, Grace Y. Wang, Vasileios Stavropoulos, Lee Kannis-Dymand, Daria J. Kuss

**Affiliations:** 1 International Gaming Research Unit and Cyberpsychology Research Group, Nottingham Trent University, Nottingham NG1 4FQ, UK; 2 Centre of Excellence in Responsible Gaming, University of Gibraltar, Gibraltar GX11 1AA, UK; 3 NTU Psychology, Nottingham Trent University, Nottingham NG1 4FQ, UK; 4 School of Psychology and Wellbeing, University of Southern Queensland, Darling Heights, QLD 4350, Australia; 5 Centre for Health Research, University of Southern Queensland, Darling Heights, QLD 4350, Australia; 6 College of Health and Biomedicine & Institute for Health and Sport, Victoria University, Footscray, VIC 3011, Australia; 7 School of Health, University of the Sunshine Coast, Sippy Downs, QLD 4556, Australia

**Keywords:** gaming disorder, substance use, co-occurrence, coping, latent profile analysis

## Abstract

*Background:* Evidence suggests that gamers can have varying experiences of disordered gaming behaviours due to coping mechanisms and how they can act as risk or protective factor in the development and/or maintenance of disordered behaviours. A particular area of interest is how this may manifest across different countries. Understanding the interplay of these potential risk and protective factors within different countries will aid identifying and preventing disordered behaviours. *Methods:* Three cohorts were recruited from Australia, New Zealand, and the United Kingdom. Each cohort was required to complete a battery of psychometric scales exploring problematic behaviours, problematic substance use, co-occurrence, coping styles, and personality. A latent profile analysis was conducted to examine the differences between cohorts and further investigated with additional analyses. *Results:* The findings suggested that a minority of gamers were affected by gaming disorder, and there appeared an at-risk cohort who utilise gaming as a maladaptive coping strategy. Other accompanying potentially addictive behaviour or substance use may be exacerbated as a result, the manifestation of which can be influenced by cultural elements. *Conclusions:* When considering gamers from countries which hold similar views, it is important to be cognisant of the variations found in the manifestations of disordered gaming and accompanying potentially addictive behaviours. This will allow for a more precise identification of at-risk behaviours, which will result in more favourable treatment outcomes for those who are considered at-risk or high-risk individuals.

## 1. Introduction

Approximately 2.9 billion individuals play videogames worldwide [1]. In some Western countries—such as Australia (AU) and New Zealand (NZ)—over 90% of households own a videogame device, and two-thirds of the population play videogames regularly [2,3]. This is not isolated to the Western countries of Australasia, but is also seen in the United Kingdom (UK), which has the largest videogame market in Europe and the sixth-largest videogame market worldwide [4]. Consequently, to better understand the positive and negative aspects of this rapidly growing leisure activity, research into gaming has been increasing at a rapid pace.

Understanding the way in which videogames can positively impact those who play them is important. Research has suggested that moderated videogame play can result in improved interpersonal skills, increased positive affect, and positive mental wellbeing [5,6]. Moreover, it has been shown to increase resilience and coping among adolescents [7]. However, it is also important to understand the association between poor mental health and videogaming and to provide insight concerning the intrinsic and extrinsic factors that may precipitate or perpetuate gaming disorder (GD) outcomes [6]. A growing body of research associates excessive gaming with poor mental health [8] and other negative consequences [9]. Therefore, there is a need to improve screening, assessment, definition, and treatment of disordered gaming.

### 1.1. Gaming Disorder

Based on growing research, the American Psychiatric Association [10] included internet gaming disorder (IGD) as a behavioural addiction (warranting further investigation) in the appendix of the latest (fifth) edition of the *Diagnostic and Statistical Manual of Mental Disorders* (DSM-5). In addition, the World Health Organization [11] has for the first time officially recognized ‘gaming disorder’ (GD) as a disorder with addiction-like properties in the eleventh revision of the *International Classification of Diseases* (ICD-11). The conceptualisations of each of these constructs overlap significantly. More specifically, the similarities of each indicate that (I)GD comprises a persistent engagement with videogames, to the point it cannot be willingly stopped and impairs individuals’ everyday functioning. It is worth noting that the constructs of IGD and GD have undergone conceptual evolution prior to inclusion in the diagnostic manuals (DSM-5, ICD-11), with several other terms used to describe problematic and disordered gaming (e.g., pathological videogaming [12]). Therefore, to maintain consistency, the term ‘GD’ here refers to the clinically defined measures of the disorder as defined by the DSM-5/ICD-11 and the term ‘disordered gaming’ will be used to describe a range of similar and/or overlapping addictive, compulsive, and/or problematic gaming behaviours which do not fit the clinically defined GD construct. 

Several studies have associated disordered gaming with mental disorders, such as anxiety [13], depression [14], substance abuse (e.g., alcohol use disorder (AUD) [15,16]), and personality disorders [17]. Findings such as these have stimulated interest into the ways that GD may influence these factors. There have been concerns that for some individuals, playing videogames may be inherently addictive, or that pre-existing vulnerabilities (e.g., anxiety and depression) increase the likelihood of GD behaviours [18,19]. There has been debate in the field as to the validity of the GD diagnosis, with scholars citing the lack of clinical populations, the heterogeneity of the gaming experience, and the risk of pathologizing ordinary gaming behaviour [20,21,22,23]. Indeed, there appears to be evidence that suggests potentially addictive behaviours can be experienced differently by individuals over time. For example, in a longitudinal gambling disorder study, the researchers found that emotionally vulnerable and impulsive gamblers transitioned between the identified gambling subtypes, indicating that these two gambling subtypes had different experiences of problematic gambling [24]. Moreover, it is possible that disordered gaming behaviours are experienced differently across gamers. For example, within massively multiplayer role-playing games (MMORPGs), gamers control an avatar (i.e., virtual character) and whether they have high levels or low levels of interaction with their virtual avatar can influence the development of disordered gaming. In addition, it has been shown that gamers with different levels of social engagement may also present different risks of disordered gaming behaviour—illustrating that the experience of disordered gaming can vary from gamer to gamer in a number of ways [19,25,26]. 

Disordered behaviours, such as GD, are not created in a vacuum, and can be considered as a collection of complex processes with multiple facets that vary across different behaviours [27,28]. This has been considered a fundamental perspective across the addiction field [29], and within this process, personality traits (e.g., instability, impulsivity) have also been shown to play a part [30,31]. More specifically, research suggests that personality factors, such as low emotional stability, low agreeableness, and low conscientiousness are associated risk factors within addiction, including gaming disorder [32] and substance abuse [33]. It has also been suggested that these personality traits can vary across different disordered behaviours [29]. For example, gambling is positively associated with low emotional stability (i.e., neuroticism [34]), while gaming is negatively associated with extraversion [35]. However, at times the relationship between personality and disordered behaviours can be ambiguous, such as extraversion within problematic social media use, and excessive studying behaviours [29]. Therefore, understanding the personality nuances within these behaviours may help give a more holistic view of associated risk and protective factors. In addition, it has also been suggested that different coping strategies may be a result of, or partially attributed to, a diverse set of risk and protective factors among gamers [36,37]. It is important to understand how coping may vary across different gamers and how this may also be influenced by personality factors.

### 1.2. Coping

Coping can be defined as the cognitive and behavioural response that occurs when an individual processes and manages stressful life events and emotions [38]. The association between gaming and coping has been considered by various scholars [37]. Among these, there have been three main domains that have been explored: problem-focused coping, emotion-focused coping, and dysfunctional coping [39,40,41]. In brief, problem-focused coping involves an active attempt to provide and implement solutions to reduce the life stressor (e.g., planning), and emotion-focused coping involves an attempt to engage and manage the unwanted negative emotions caused by the life stressor (e.g., humour [39]). Finally, dysfunctional coping involves an attempt to avoid or disengage the unwanted negative emotions or life stressors (e.g., denial [40,41]). 

A number of scholars have explored coping and its association with disordered gaming [36,41], with some pointing to a potential link. For instance, those with dysfunctional coping strategies tend to have an increased risk of psychopathology (e.g., depression, anxiety [42]), disordered behaviour, disordered substance use [37], and high neuroticism [43]. In a recent study conducted among a sample of Polish students, researchers found that participants who utilise media-focused coping strategies (e.g., gaming) to regulate their everyday life stressors appeared to have a higher risk of maladaptive coping behaviours. They concluded that dysfunctional coping strategies can exacerbate GD symptomology [41]. This finding is supported by a study which examined over 800 secondary students, whose disordered gaming behaviour was significantly associated with denial and behavioural disengagement—two coping styles which fall under the broader dysfunctional coping strategy domain [36]. This suggests that gamers play videogames in order to destress and to escape, and therefore some scholars suggest that this may fulfil a compensatory function in supporting individuals to cope with psychosocial problems [44]. 

These findings have led researchers to posit that disordered gaming may be, in part, better characterised as a manifestation of maladaptive coping strategies which have the potential to be exacerbated by other psychosocial issues [36]. For example, disordered gaming has been frequently associated with a pattern of escapism among individuals with depression [14]. Consequently, the continued use of gaming to escape may become an over-relied upon strategy resulting in negative long-term consequences with respect to the ability to cope with subsequent situations in which the primary coping strategy is not available. This may encourage an individual to seek other maladaptive habits in order to cope [37]. This may, in turn, further exacerbate psychopathological disorders such as depression and anxiety [41]. Indeed, research suggests that disordered gaming, like other behavioural disorders (e.g., gambling), can co-occur with problematic behaviours or substance use [37]. Additionally, disordered gaming appears to interact and/or co-occur with other conditions, which may result in complications for both risk assessment and diagnosis [37]. Therefore, it is important to consider the impact gaming has on individuals who have (or are at-risk of) disordered behaviours [19].

### 1.3. Co-Occurrence of Addictive Behaviours

Co-occurrence occurs when two or more potentially addictive behaviours (behavioural and/or substance-related) are engaged in concurrently or in close temporal proximity. In a recent review of co-occurrence of GD with other disordered behaviours, it was found that the presence of co-occurring disordered behaviour—or substance use—exacerbated symptomology of GD [37]. For example, Na et al. [45] found that South Korean adults who engaged in both problematic alcohol use and problematic gaming exhibited higher cigarette smoking rates than those who engaged in problematic alcohol use or problematic gaming alone. This was also supported by Ream et al.’s study [46], who investigated an American sample of adult gamers who had significant correlations between substance use and problematic videogame use, noting that the substances were often consumed while gaming.

The overlap in potentially addictive behaviours appears to create a cycle of reciprocity [47,48,49], in which mutual exacerbation occurs between two or more problematic behaviours. This may explain why individuals who experience more than one disordered behaviour display poorer outcomes in relation to physical and mental wellbeing [14,49,50]. Consequently, the mutual exacerbation can create complications within clinical symptomology—confounding accurate assessment, diagnosis, and treatment of psychiatric disorders [51]. Similarly, GD may exacerbate existing addictive behaviours (e.g., substance use), causing symptomology of each behaviour to alternate—and therefore impacting treatment efficacy [52]. Therefore, clinicians and scholars should be aware of the way in which addictive behaviours may impact or enforce various aspects of a presenting disorder (e.g., GD), and consider how co-occurrence and contextual factors (e.g., coping strategies) may impact the onset, course, and outcomes of interventions.

Although there is an association between coping, co-occurrence, and GD, additional research is needed into how these may be influenced across varying cultural contexts [46]. There is research to suggest that co-occurring disordered behaviour or substance use can vary based on geographical location [37], and that one’s country of origin can moderate disordered gaming [53]. Consequently, the field would benefit from the exploration of the cultural nuances found in co-occurring disordered behaviours, coping strategies, and gaming behaviour [54]. 

### 1.4. Gaming and Culture

Scholars have consistently asserted that culture can impact psychopathology [54,55,56]. Further research indicates that it can impact the experience and understanding of psychosocial, addictive, and psychopathological disorders [8]. Moreover, several studies have explored cross-cultural variations in videogame playing behaviour, suggesting that the culture context the individual is based in can impact GD severity [6,57]. Therefore, it is important that the field develops an understanding of how GD may be experienced across differing regions, in an attempt to better understand the development and maintenance of disordered gaming behaviours [53,54,58]. 

In a broader context, culture might be described as patterns of behaviour that are explicitly and implicitly acquired and are communicated through symbols and/or practices, which are shared by those who accompany a collective/social identity [59]. There has been research conducted into the way cultures accept and interreact with technology, with Hofstede’s [60,61] proposed cultural dimensions being reliable in the field of information technology [62,63]. Hofstede’s cultural dimensions [60,61] attempt to categorise dominant cultures by systematically differentiating them from each other across six dimensions: power/distance, femininity/masculinity, uncertainty/avoidance, individualism/collectivism, long-term orientation, and indulgence. The present study focuses on cultures which present with high individualism as opposed to collectivism through the lens of Hofstede’s proposed cultural dimensions. Individualistic societies tend to be more loosely socially connected, and individuals in these cultures tend to identify as an ‘I’ rather than a ‘we’. Consequently, they tend to prioritise themselves and their immediate families rather than those with whom they are unfamiliar [60,61]. 

Hofstede’s [60,61] cultural dimensions provide a general understanding of the way in which a national culture expects, perceives, and assesses the values of its members. However, the theory has been criticised because it oversimplifies national culture—neglecting multicultural trends and individual differences found within each culture [64]. Nevertheless, there have been a number of studies which have considered cross-cultural comparisons in the GD literature [54,56], with a specific focus on the dichotomy between individualistic and collectivist cultures. Consequently, the nuance of either culture is not explored in depth. This is an important factor to consider because research suggests that within individualistic cultures, substance use and behaviours can differ depending on the geographical location of the culture [37]. For example, the United Nations’ ‘World Drug Report 2020′ [65] estimates that 1.3% of Australians use amphetamines, while England (including Wales) and NZ have rates of 0.6% and 0.8%, respectively. In addition, estimates of problematic behaviours and their co-occurrence can also differ across culturally diverse groups of individuals [37,66]. Therefore, understanding the way in which cultural context may influence problematic substance use, behaviours, and subsequent co-occurrence is of particular importance. 

Due to the intra-cultural differences found in behaviour and substance use, factors which influence coping styles (e.g., denial, escapism) within each country may also vary. This impacts the way individuals use videogames in relation to life stressors and the potential of co-occurring problematic use. A recent review by Burleigh et al. [37] reported four studies which considered coping in relation to disordered gaming. This demonstrates the need for further empirical evidence to better understand how individuals may utilise coping in a gaming context as a risk or protective factor against co-occurrence. Therefore, to gain a better understanding of how cultural dimensions may apply to disordered gaming, and to address the need of nuanced investigation of intra-cultural dimensions, in the present study, three countries considered individualist were explored [67], with a focus on gaming, personality factors, coping styles, and disordered substance use and/or behaviours and their potential co-occurrence.

### 1.5. The Present Study

There has been evidence to suggest that gamers can have varying experiences of problematic gaming behaviours [25,26]. These varying experiences have been suggested to be due to coping mechanisms and how they can act as risk or protective factor for the development and/maintenance of disordered behaviours [36]. Furthermore, coping mechanisms can also shed light on the way an individual interacts and or engages in disordered behaviours—with research suggesting that dysfunctional coping strategies can result in exacerbating disordered behaviours through a cycle of co-occurrence and reciprocity [41]. A particular area of interest is how this may manifest across different countries. A number of studies have considered the dichotomy between individualistic and collectivist countries [54,55,56], focusing on the individualistic/collectivist attributes (e.g., competitiveness) that citizens in each country possess and how they differ. However, in doing so, they have overlooked the nuanced differences in disordered behaviours, personality factors, coping strategies, and the potential of co-occurrence found across similar countries in very different geographical locations [37]. This is an important facet to consider because understanding the interplay of these potential risk and protective factors within each of these countries will aid identifying and preventing disordered behaviours. 

Researchers have explored a number of these facets (e.g., gaming and coping [37]) using a variable-centred approach. This is an approach which provides specific information on the importance of each factor on the outcome variable [68]. However, these methods can be somewhat flawed when the assumption of homogeneity is applied to the sample [69]. Therefore, the present study considers a person-centred approach which is suited to examining the similarities and differences across participants, while considering how variables interact with one another [70]. This approach has a number of advantages because it can (i) assess whether distinct groups of individuals can be identified through naturalistic grouping of factors; (ii) offer complex combinations among all possible factors at all possible levels of each factor; and (iii) be clinically appropriate because decisions concerning assessment and treatments often focus on the individual rather than on the variable or factor [71]. In conjunction with the person-centred approach, the present study utilises latent profile analysis (LPA) to identify groups of individuals within each country that have similar profiles for multiple dimensions of psychopathology and disordered behaviours. LPA is used to define unobserved subgroups based on observed variables without specifying the number of profiles in advance. Therefore, it is believed to be a more appropriate method to address research questions that are exploratory in nature and to understand the diversity and complexity of multiple risk factors within psychopathology [72].

Consequently, the present study seeks to identify profiles of individuals characterized by unique patterns of disordered behaviours (e.g., gaming, substance use, etc.), personality factors (e.g., neuroticism), co-occurrence, and coping strategies across individualised countries. It is hypothesised that (i) a profile with higher co-occurrence across all disordered behaviours will be identified (H_1_); (ii) a profile with risk of disordered behaviours will be identified (H_2_); (iii) dysfunctional coping strategies, low agreeableness, low emotional stability, and low conscientiousness will be strongly associated with the profiles that have higher scores on disordered behaviours (i.e., behavioural and substance use variables), and least strongly associated with profiles with low risk of disordered behaviours (H_3_); and (iv) a profile of disordered behaviours differentiating between countries will be identified (H_4_). 

## 2. Materials and Methods

### 2.1. Participants and Procedure

Participant data were collected from September 2019 to September 2021 across four universities in three countries: The UK (Nottingham Trent University), NZ (Auckland University of Technology), and AU (Victoria University and the University of the Sunshine Coast). Flyers and online advertisements were used around each campus. The inclusion criteria for this sample were (i) being aged 18 years or over; and (ii) currently residing in the country where the survey was taken (i.e., UK, NZ, AU). If individuals met this criterion, they were able to participate in the anonymous online survey. Consequently, each cohort was recruited using convenience sampling. The UK sample comprised of 561 participants, including 416 women (M*_age_* = 19.8 years; *SD* = 1.47) and 139 men (M*_age_* = 20.7 years; *SD* = 2.68 years), aged between 18 and 36 years (M*_age_* = 20 years; *SD* = 1.88). The NZ sample comprised 170 participants, including 88 women (M*_age_* = 26.6 years; *SD* = 8.83) and 80 men (M*_age_* = 25.6 years; *SD* = 9.07 years), aged between 18 and 65 years (M*_age_* = 26.1 years; *SD* = 8.89). Lastly, the AU sample comprised 1185 participants, including 428 women (M*_age_* = 28.2 years; *SD* = 10.2) and 772 men (M*_age_* = 28.8 years; *SD* = 8.94 years), aged between 18 and 64 years (M*_age_* = 28.5 years; *SD* = 9.35). The survey took approximately 20 min to complete. Additional sociodemographic information is shown in Table 1. In order to minimise risk to participants, each individual was given information prior to the study, including data use, potential risks, and benefits, along with their right to withdraw from the study until data analysis and contact details to the research team. Participants were fully informed on what to expect throughout the survey and were made aware at any point they could terminate the online survey by closing their browser. Due to the study containing information about disordered behaviours, participants may have experienced distress caused by some survey items. Therefore, information related to counselling services was also provided (e.g., *Samaritans* in the UK, *Headspace* in AU, the university’s free counselling service in NZ). The study was approved by each of the university’s ethics committees. 

### 2.2. Measures

#### 2.2.1. Nine-Item Internet Gaming Disorder Scale–Short Form (IGDS9-SF)

The nine-item IGDS9-SF [72] was used to assess the severity of GD symptoms (over the past 12 months) by examining an individual’s offline and online behaviours. Items include *“Do you systematically fail when trying to control or cease your gaming activity?”* and *“Have you jeopardized or lost an important relationship, job or career opportunity because of your gaming activity?”.* Participants respond to each item on a five-point scale from 1 (*never*) to 5 (*very often*). The final GD score is calculated by summing up the individual’s answers and ranges from 9 to 45, with higher scores indicating higher severity of gaming disorder behaviours. The scale has been shown to be a reliable measure with a Cronbach’s α of 0.88 [72]. In the present study, the scale showed very good reliability (Cronbach’s α = 0.88). 

#### 2.2.2. Problem Gambling Severity Index (PGSI)

The nine-item PGSI [73] was used to assess problem gambling over the past 12 months (e.g., *“Have you gone back on another day to try to win back the money you lost?”*). Participants respond to items on a four-point scale ranging from 0 (*never*) to 3 (*always*). The total score is obtained by summing up each of the answers given and can range from 0 to 27, with higher scores indicating greater gambling severity. The final score relates to one of four gambling domains: non-problem gambler = 0; Low-risk gambler = 1–2; Moderate-risk gambler = 3–7; Problem gambler = 8 or above. This has been shown to be a reliable scale with a Cronbach’s α of 0.76 [73]. In the present study, the scale showed excellent reliability (Cronbach’s α = 0.94).

#### 2.2.3. Nine Item Internet Disorder Scale–Short Form (IDS9-SF)

The nine-item IDS9-SF [74] was used to assess problematic internet use behaviours over the past 12 months (e.g., *“Do you feel more irritability, anxiety and/or sadness when you try to either reduce or stop using the internet?”*). Responses are scored on a five-point scale ranging from 1 (*never*) to 5 (*very often*). The final score is calculated by adding each item score which gives a total score ranging from 9 to 45. Higher scores indicate a greater severity of disordered internet use. The IDS9-SF has been shown to be a reliable scale with a Cronbach’s α of 0.96 [74]. In the present study, the scale showed very good reliability (Cronbach’s α = 0.89).

#### 2.2.4. The Bergen Social Media Addiction Scale (BSMAS)

The BSMAS [75] is an adapted version of the Bergen Facebook Addiction Scale (BFAS) [76] and includes six items assessing addictive social media use (e.g., *Facebook, Instagram, Twitter*) in the past 12 months. Each item reflects a core addiction element (e.g., withdrawal: *“How often have you become restless or troubled if you have been prohibited from using social media?”*) and is scored on a five-point scale ranging from 1 (*very rarely*) to 5 (*very often*) and can have a total score between 6 and 30. A higher score indicates a greater risk of social media addiction. The BSMAS has very good reliability (Cronbach’s α = 0.88 [75]). In the present study, the scale showed very good reliability (Cronbach’s α = 0.89).

#### 2.2.5. Bergen-Yale Sex Addiction Scale (BYSAS)

The six-item BYSAS [77] was used to assess participants’ problematic sexual activity over the last 12 months (e.g., *“How often … have you spent thinking about sex or masturbation?”*). Each response is scored on a five-point scale, with scores ranging from 0 (*very rarely*) to 4 (*very often*). To obtain the total score, the scores on each item are summed. The total score can range from 0 to 24, with a higher total score indicating a greater risk of sex addiction. The BYSAS has been found to be a reliable scale with a Cronbach’s α of 0.82 [77]. In the present study, the scale showed very good reliability (Cronbach’s α = 0.84).

#### 2.2.6. Bergen Shopping Addiction Scale (BSAS)

The seven-item BSAS was used to assess for problematic shopping behaviour over the past 12 months [78]. Participants respond to each item (e.g., “I think about shopping or buying things all the time”) on a five-point Likert scale from 0 (completely disagree) to 4 (completely agree). The final score is calculated by summing up the individuals’ answers and ranges from 0 to 28, with higher scores indicating greater risk of shopping addiction. The BSAS has been found to be a reliable scale with a Cronbach’s α of 0.87 [78]. In the present study, the scale showed excellent reliability (Cronbach’s α = 0.90). 

#### 2.2.7. Exercise Addiction Inventory–Revised (EAI-R)

The six-item EAI-R was used to assess addictive exercise over the past 12 months [79]. Participants respond to each item (e.g., *“Over time I have increased the amount of exercise I do in a day”*) on a six-point Likert scale from 1 (*strongly disagree*) to 6 (*strongly agree*). The final score is calculated by summing up the individual’s answers and ranges from 6 to 36, with higher scores indicating greater risk of exercise addiction. The EAI-R has been found to be a reliable scale with a Cronbach’s α of 0.90 [79]. In the present study, the scale showed very good reliability (Cronbach’s α = 0.85).

#### 2.2.8. Cigarette Dependency Scale–5 (CDS)

The five-item CDS-5 [80] was used to assess the degree to which participants were dependent on cigarettes. Each item is scored on a five-point scale and assesses their cigarette use (e.g., *“Please rate your addiction to cigarettes on a scale of 0 to 100?”*) and habits (e.g., *“Usually, how soon after waking up do you some your first cigarette?”*). Questions 1 to 3 are open questions (e.g., “On average, how many cigarettes do you smoke per day?”) where a participant can write their response. The response is then converted into a five-point scale (e.g., “8 cigarettes per day” equates to a score of 2 [6–10 cigarettes per day]). Questions 4 and 5 require typical responses with scores ranging from 1 (*totally disagree*) to 5 (*fully agree*). Questions 3 and 4 are both reverse coded, where the lower point is scored as 5 and the higher point is scored as 1 (e.g., *“For you, quitting smoking would be “Impossible”* [5] to *“Very Easy”* [1]). The final score is calculated by summing up the answers and ranges from 5 to 25, with higher scores indicating higher severity of cigarette dependant behaviours. The CDS-5 has been found to be a reliable scale with a Cronbach’s α of 0.83 [80]. However, in the present study, the scale showed lower reliability (Cronbach’s α = 0.68).

#### 2.2.9. Alcohol Use Disorder Identification Test (AUDIT)

The ten-item AUDIT [81] was used to assess alcohol consumption, drinking behaviours, and alcohol-related problems (e.g., *“How often do you have six or more drinks on the one occasion?”*) over the past 12 months. Items 1 to 8 are rated on a five-point scale, which are scored from 0 (e.g., *“Never”*) to 4 (e.g., *“Daily or almost daily”*), whereas Items 9 and 10 are rated on a three-point scale and are scored as 0 (*“No”*), 2 (*“Yes, but not in the past”*), and 4 (*“Yes, during the past year”*). The total score comprises the summing of each of the selected item scores. The total score can range from 0 to 40. A score of 8 or more indicates hazardous drinking. A score of 13 or more in women, and 15 or more in men, may indicate alcohol dependence. The AUDIT has demonstrated good reliability. For example, in a systematic review by Meneses-Gaya et al. [82] across ten studies the average Cronbach’s alpha was 0.80. In the present study, the scale showed very good reliability (Cronbach’s α = 0.87).

#### 2.2.10. Drug Abuse Screen Test—10 (DAST)

The ten-item DAST-10 [83] was used to assess drug use behaviours in the past 12 months (e.g., *“Do you feel bad or guilty about your drug use?”*). Each item is rated on a dichotomized scale (yes/no answers). Each “Yes” answer is scored with 1, while each “No” answer is score with 0—except for Question 3 for which a “No” is scored with 1 while “Yes” is scored with 0. The total score ranges from 0 to 10: 0 = no problems; 1–2 = low problems; 3–5 = moderate problems; 6–8 = substantial problems; 9–10 = severe problems. In a systematic review on the psychometric properties of the DAS, Yudko, Lozhkina, and Fotus [84] reported it to be a reliable measure with multiple studies citing a Cronbach’s α of over 0.90. In the present study, the scale demonstrated very good reliability (Cronbach’s α = 0.83). 

#### 2.2.11. Brief Coping Orientation to Problems Experienced (Brief-COPE)

The 30-item Brief-COPE [85] was used to assess coping behaviours individuals employed when experiencing stressful situations. Participants are asked to think about a recent stressful event in their life and how they coped within that situation. The Brief-COPE is rated on a four-point scale: 1 (*I haven’t been doing this at all*), 2 (*I’ve been doing his a little*), 3 (*I’ve been doing this a medium amount*), and 4 (*I’ve been doing this a lot*). The Brief-COPE has a total of 15 two-item subscales (e.g., self-distraction, substance use, humour). The subscale scores are then added together to give a score ranging from 2–8. A higher score on the subscale represents a higher utilisation of the related coping behaviour. These smaller subscales form a super-ordinate domain coping style. These are emotion-focused coping (EFCope; scoring from 10 to 40; Cronbach’s α of 0.83), dysfunctional coping (DCope; scoring from 12 to 48; Cronbach’s α of 0.82), and problem-focused coping (PFCope; scoring from 6–24; Cronbach’s α of 0.76). The internal consistency estimates of the current study reflect the previously reported estimates in Carver’s [85] paper (which ranged between 0.50 to 0.90), thus we consider the psychometric properties to be acceptable.

#### 2.2.12. Ten Item Personality Inventory (TIPI)

The 10-item TIPI [86] was used to briefly assess personality traits. The TIPI assesses extraversion, agreeableness, conscientiousness, emotional stability, and openness to experiences. Participants are asked to agree or disagree with a statement using a seven-point Likert scale from 1 (*disagree strongly*) to 7 (*agree strongly*). Each even number item (e.g., 2, 4, 6, etc.) is reverse scored and then the items are paired off into each of the five subscales. These two items are then averaged to give the total score of that subscale with higher scores indicating more pronounced personality traits. The original TIPI [86] showed low-to-moderate Cronbach’s alphas (α = 0.40–0.68), which is a common range within short scales [87]. The present study had a Cronbach’s α ranging between 0.29 (Agreeableness) to 0.79 (Extraversion). As such, to better investigate the reliability of the present scale a McDonald’s coefficient omega was calculated at 0.52, 95% CI [0.46–0.58], indicating adequate internal consistency. 

### 2.3. Data Analysis

First, the missing data imputation was calculated for the study variables and followed by descriptive statistics. In order to test the hypotheses, an LPA was conducted, a strategy which is appropriate for continuous indicators [88]. The LPA investigates whether relatively homogeneous groups (i.e., profiles) can be identified based on observed values [89]. This shows whether structural groups exist in the data, where participants show similarities and differences with each other.

To examine whether and to what extent disordered behaviours differed across individualised countries, an LPA was conducted in *Rstudio* using *tidyLPA* [90]. For the LPA, disordered behaviours and substance use, personality, and coping domain variables were included. More specifically, scores of disordered gaming, internet use, social media, shopping, gambling, sex, exercise, drug use, alcohol use, and cigarette use were included, alongside scores on the five domains of personality (i.e., extraversion, agreeableness, conscientiousness, emotional stability, and openness to experience) and the three sub-domains of coping (i.e., problem-focused coping, emotion-focused coping, and dysfunctional coping). Within LPA, a range of models are predicted, and within these models an increasing number of profiles are tested. The Bayesian information criterion (BIC) and Akaike information criteria (AIC) indicate the fit of the model used, with the lower numbers indicating a better fit [91]. Entropy indicates how well the model classifies participants into different profiles without overlap in or exclusion from other profiles. As such, lower entropy scores indicate that participants can be classified into more than one profile; therefore, entropy scores of 0.80 and over are recommended [91]. Lastly, the bootstrap likelihood ratio test (BLRT) indicates whether models with one additional profile outperform the previous model.

To further validate the assessment of differences between the classes and to investigate the extent of these differences, a collection of multivariate analyses of variance (MANOVAs) was used between the identified profiles of each cohort and their effect sizes [92,93]. Following this, a pairwise comparison was conducted on the appropriate profiles and variables to investigate the specific differences between the selected profiles. 

## 3. Results

The LPA was conducted using the equal variances and covariances fixed to zero model, which is a class-invariant parameterization (CIP) model. While this model is highly constrained, it is also parsimonious. The profiles are estimated so that the variables’ variances are the same for each profile. Therefore, the relationships between the variables are not calculated. In addition, covariances are constrained to zero [90]. This model demonstrated a better fit for the data and interpretability when compared to other LPA models. As seen in Table 2, the BIC suggested a four-model class across each of the cohorts. The BIC is considered the most reliable fit statistic in LCA [91]. Therefore, a four-profile model was used. It is also important to note the four-profile model had adequate entropy (i.e., above the cut-off of 0.80 [91]), indicating that participants were assigned to profiles effectively. 

**Figure 1:**
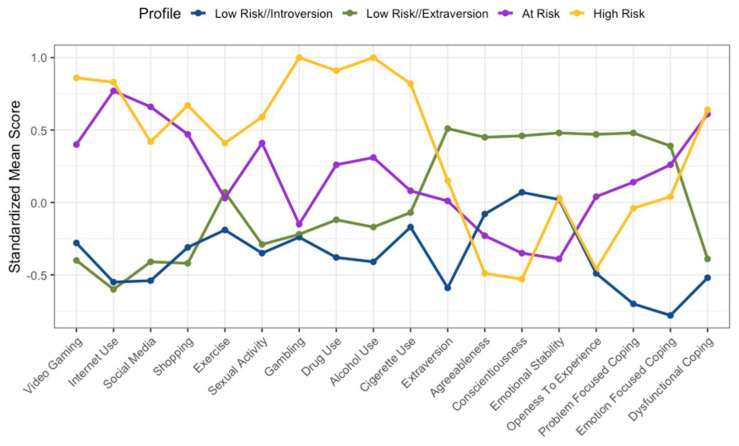
Standardized mean score graph of the UK cohort.

**Figure 2:**
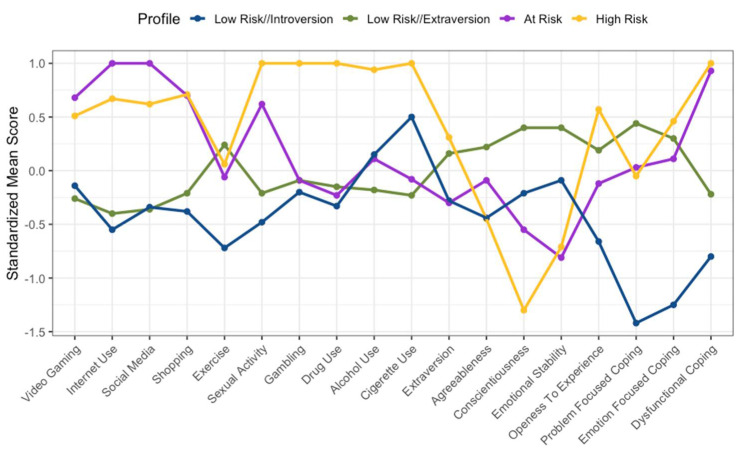
Standardized mean score graph of the New Zealand (NZ) cohort.

**Figure 3:**
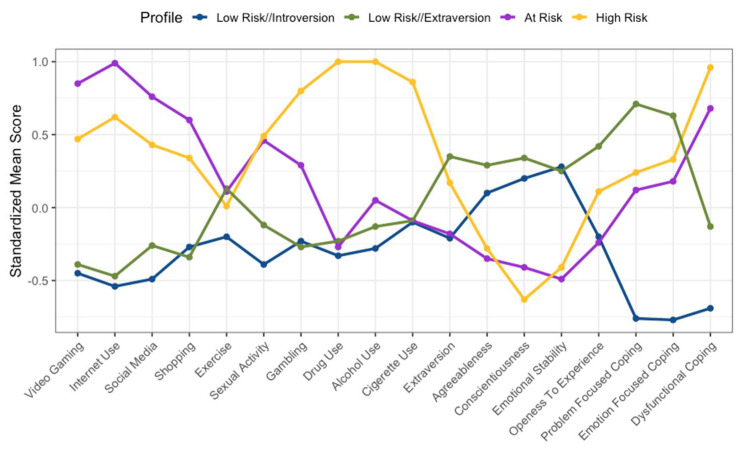
Standardized mean score graph of the AU cohort.

Figure 1, Figure 2 and Figure 3 show a graphical representation of each of the three cohorts and their four-profile model. The *x*-axis provides the names of each of the behaviours, personality factors, and coping style variables. The *y*-axis provides the standardised mean score of each profile in relation to each variable. Each cohort appeared to exhibit two profiles that averaged higher on disordered behaviours and dysfunctional coping strategies, and two profiles that averaged lower across disordered behaviours and dysfunctional coping strategies. Therefore, the two profiles which displayed higher scores on the disordered behaviour measures and on the dysfunctional coping strategies were classified as risk profiles. The two risk profiles were classified as ‘at-risk’ (i.e., the profile which typically demonstrated high disordered behaviour scores and lower substance use and dysfunctional coping scores in comparison to the ‘high-risk’ profile) and ‘high risk’ (i.e., the profile which demonstrated consistent high disordered substance use scores and a higher dysfunctional coping score, in comparison to at-risk). This process was done visually and was dependent on the scores obtained, and therefore a somewhat subjective choice; nevertheless, it is warranted in order to communicate the data effectively. The remaining profiles were classified as ‘low-risk’ profiles. These were spilt into low-risk extraversion and low-risk introversion, as each cohort demonstrated a low-risk profile with higher extraversion and lower extraversion (i.e., introversion). However, in line with the hypotheses, the present paper will consider the at-risk and high-risk profiles. 

### 3.1. UK Cohort

Figure 1 shows four profiles found in the UK cohort. The at-risk profile was the larger of the two risk profiles and comprised 220 participants (38.86% of UK cohort), while the high-risk profile comprised 26 participants (4.59% of UK cohort). In the UK Cohort, it can be seen that the high-risk profile demonstrated a consistently higher standardised difference from the sample mean (z) than the at-risk profile, except on social media use (high-risk: *z* = 0.42; at-risk: *z* = 0.66). In addition, individuals with the high-risk profile scored lower on personality factors (bar emotion stability; high-risk: *z* = 0.03; at-risk: *z* = −0.39) and scored lower on problem-focused (*z* = −0.04) and emotion-focused (*z* = 0.04) coping strategies when compared to the sample mean of the at-risk profile (*z* = 0.14, *z* = 0.26, respectively). Both the at-risk and high-risk profiles demonstrated a clear reliance on dysfunctional coping strategies (*z* = 0.61, *z* = 0.64, respectively). Further details in relation to the UK cohort can be seen in Table 3. 

### 3.2. New Zealand Cohort

Figure 2 shows the four profiles found in the NZ cohort. As in the previous analysis, there was a noticeable difference seen between the at-risk and high-risk profiles. The at-risk profile comprised 34 participants (20% of NZ cohort), while the high-risk profile comprised 10 participants (5.88% of NZ cohort). Within the NZ cohort, the at-risk profile demonstrated three higher standardised scores from the sample mean across gaming (z = 0.68), internet use (*z* = 1.00), and social media use (*z* = 1.00) in comparison to the high-risk profile (*z* = 0.51, 0.62, 0.67, respectively). As with the UK cohort, the high-risk cohort demonstrated a higher standardized difference from the sample mean across substance use (ranging from *z* = 0.96–1.00), whereas the at-risk profile demonstrated lower scores (ranging from *z* = −0.23 to 0.11). In addition, the high-risk profile had a low conscientious score (*z* = −1.30) and a high openness to experience score (*z* = 0.57), in contrast to the at-risk profile (*z* = −0.55, −0.12, respectively). Furthermore, both the at-risk and high-risk profiles demonstrated a high standardized difference from the sample mean (*z* = 0.93, *z* = 1.00, respectively). Further details in relation to the NZ cohort can be seen in Table 4.

### 3.3. Australian Cohort

Figure 3 shows the four profiles found in the AU cohort. As in the previous two analyses, there was a noticeable difference between the at-risk and high-risk profiles. The at-risk profile comprised 314 participants (26.49% of AU cohort), while the high-risk profile comprised 115 participants (9.70% of AU cohort). As with the two previous cohorts, the AU cohort demonstrated a consistently high standardised score from the sample mean in both the at-risk and high-risk profiles. The AU cohort’s at-risk profile demonstrated higher standardised scores from the sample mean across gaming (z = 0.85), internet use (z = 0.99), social media use (z = 0.76), and shopping (z = 0.60). The high-risk profile consistently had a higher standardised score from the sample mean across substance related behaviours (ranging from z = 0.86 to z = 1.00). In regard to personality variables, both the at-risk (z = −0.41) and high-risk profiles (z = −0.63) scored quite low on conscientiousness and scored quite high in dysfunctional coping strategies (z = 0.68, z = 0.96, respectively). Further details in relation to the AU cohort can be seen in Table 5.

### 3.4. At-Risk Profile across Cohorts

To investigate if the at-risk profiles differed significantly from each cohort, a one-way multivariate analysis of variance (MANOVA) was performed. A MANOVA was used to determine the difference of the at-risk cohort group on the behavioural (e.g., videogaming, internet use), substance (i.e., drug, alcohol, and cigarette use), personality (e.g., extraversion, agreeableness), and coping variables (i.e., problem-focused, emotion-focused, and dysfunctional coping). The three groups investigated were (i) UK (at-risk profile), (ii) NZ (at-risk profile), and (iii) AU (at-risk profile). There was a statistically significant difference between the groups on the combined dependent variables (i.e., behaviour, substance, personality, and coping), *F*[36, 1098] = 3.93, *p* < 0.001; Pillai’s trace = 0.229, partial η^2^ = 0.114. Follow-up univariate Welch ANOVAs using Hochberg correction showed there was a statistically significant group difference in scores in videogaming (*F*[2, 87.9] = 13.6, *p* < 0.001), internet use (*F*[2, 91.7] = 12.3, *p* < 0.001), gambling (*F*[2, 91.2] = 16.2, *p* < 0.001), and drug use (*F*[2, 100] = 22.9, *p* < 0.001). Games Howell pairwise comparisons (adjusted Tukey *p*-value) were then conducted between the group’s significant variables (i.e., videogaming, internet use, gambling, and drug use). Significant differences were observed (see Table 6).

### 3.5. High-Risk Profile across Cohorts

To investigate if the high-risk profiles differed significantly from each cohort, a one-way multivariate analysis of variance (MANOVA) was performed. The three groups investigated were (i) UK (high-risk profile), (ii) NZ (high-risk profile), and (iii) AU (high-risk profile). There was a statistically significant difference between the groups on the combined dependent variables (i.e., behaviour, substance, personality, and coping), *F*[36, 264) = 3.96, *p* < 0.001; Pillai’s trace = 0.702, partial η^2^ = 0.351. Follow-up univariate Welch ANOVAs, using Hochberg correction, showed that there was a statistically significant group difference in scores in gambling (*F*[2, 21] = 59.2, *p* < 0.001) and drug use (*F*[2, 100] = 21, *p* < 0.001). Games Howell pairwise comparisons (adjusted Tukey *p*-value) were then conducted between the group’s significant variables (i.e., gambling and drug use). Significant differences were observed (see Table 7).

## 4. Discussion

In the present study, firstly, the latent profiles found within the different individualistic countries of Australia, New Zealand, and the United Kingdom were explored. Secondly, these profiles were compared across the aforementioned countries to investigate the potential differences between high-risk and at-risk groups by exploring how gaming, problematic behaviour and substance use, personality factors, and coping mechanisms were reported within these individualistic countries, as opposed to the often-examined individual/collectivist dichotomy.

The findings suggest that H_1_ was partially supported. More specifically, it was found that across each cohort, there was a profile (i.e., high-risk profile) in which individuals scored consistently higher across substance use variables (i.e., drug use, alcohol use, cigarette use) with varying elevated levels of behavioural variables (e.g., gaming, social media use). Similarly, a profile with elevated levels across behavioural and substance use was also found within each profile. Individuals with this profile consistently scored lower than individuals with the high-risk profile—but higher than individuals with the low-risk profiles. Therefore, H_2_ was supported, which suggests that each cohort contains a profile which demonstrated at-risk scores, i.e., scores which were lower than the highest profile, but were higher than low-risk profiles. The profiles identified as at-risk and high-risk demonstrated consistently higher scores on dysfunctional coping strategies, and lower scores on conscientiousness. In addition, scores on emotional stability remained low in both the risk profiles and low-risk profiles, therefore partially supporting H_3_. Lastly, H_4_ was partially supported. More specifically, while the at-risk profile demonstrated significant differences between cohorts, these differences were not present across all variables.

### 4.1. Risk Profiles across Cohorts

The results of the LPA provided some support for H_4_, which suggested that within each cultural cohort, a set of unique risk factors were present. The at-risk profile made up 39.2%, 20%, and 25.5% of total UK, NZ, and AU cohorts, respectively, while the high-risk profiles made up 4.6%, 5.8%, and 9.7%, respectively.

### 4.2. Behaviour, Substance Use, and Co-Occurrence

In regard to drug use scores in the at-risk group, the UK cohort demonstrated higher scores than both the AU and NZ cohorts, while the AU and NZ cohorts did not show any significant differences. This suggests the UK cohort may have experienced higher rates of substance use. In regard to behaviours, the AU cohort demonstrated significantly higher scores on gambling and gaming, while each cohort varied in relation to disordered internet use. In regard to the high-risk sample, the UK cohort demonstrated higher scores on substance use when compared with both the AU and NZ cohorts. The AU cohort scored significantly higher on gambling than the UK cohort. These findings suggest that, within the present at-risk samples, there are nuances in how each cohort experienced behavioural and substance use disorders. This is in line with previous literature suggesting that residing in a country can impact specific risk behaviours [6,57], with the present study suggesting that this is not limited to countries that have different cultural norms. There is evidence to suggest that the country in which an individual resides can influence how they experience and manifest disordered behaviours [94]. However, as seen in the present study, this is also apparent in countries that share many intracultural similarities.

Understanding how individuals interact within their country and how their country may impact the manifestation and prevalence of disordered behaviours has important implications for at-risk individuals because the literature suggests that cultural paradigms may influence specific co-occurring problematic behaviours among at-risk individuals [94]. In the present study, the UK cohort demonstrated a higher risk in relation to substance abuse, whereas the AU cohort demonstrated a higher risk of gambling. Understanding the potential co-occurring at-risk and high-risk behaviours associated with substance use or gambling may allow researchers to better contextualise and explore issues of co-occurrence across these specific individualistic countries by considering the specific intracultural nuances. Co-occurrence is prevalent across multiple different individualistic countries [37], and research has shown that co-occurrence can exacerbate and complicate the diagnosis and treatment of clinical disorders [51]. Therefore, it is important that clinicians have access to empirical data that aids in the creation of early interventions which are tailored to at-risk groups, with a focus on the known co-occurring issues experienced within their country. While the differences in the engagement of substance use and behaviours are apparent, it should be noted that the cohorts shared similarities in both personality and coping style.

### 4.3. Personality Factors

In regard to the personality factors assessed within the at-risk cohorts, only individuals with high extraversion scores were found to score consistently higher across all three at-risk cohorts—however, it should be noted that within the UK at-risk cohort, individuals scored higher on “openness to experience” when compared to the standardised mean of the other cohorts. The remainder of the personality factors (i.e., agreeableness, conscientiousness, emotional stability, and openness to experience) were found to be negatively associated with the at-risk profile. Emotional stability was found to be the lowest scored personality trait across all three at-risk cohorts. In regard to the high-risk cohorts, it was found that individuals consistently scored lower on conscientiousness across all cohorts. Similarly, there were lower scores on agreeableness when compared to the standardised mean scores of other personality factors. There have been a number of studies which have considered personality in relation to both problematic substance use and behaviours [32,35], and the present findings suggest that within each risk profile cohort, there are consistent personality factors which are present. In other words, consistent personality factors exist across countries, and that some personality factors (e.g., low conscientiousness, extraversion) can be considered risk factors in relation to problematic behaviours and substance use [32,35].

It is well established that high levels of extraversion and low levels of conscientiousness can, and often are, used as predictors for disordered behaviours [33]. The present study also supports the established literature on disordered behaviour. However, while there appears to be consistency in a majority of the personality factors in relation to addictive behaviours, it is interesting that extraversion is consistently high despite being tested on a high majority of gamers—when previous research suggests that extraversion is typically lower in gamers [35]. This result may be due to the type of gamers surveyed. There is a large body of research which focused on individuals who play MMORPGs. Their findings suggest that more introverted individuals play these games in order to fulfil fantasies which they perhaps cannot fulfil in their offline life [14]. However, videogames have evolved significantly in the past decade; new and more competitive gaming genres (e.g., *Battle Royale* games, Multiplayer Online Battle Arena [MOBA] games) have become increasingly popular [6]. It could be that the gamers surveyed in the present cohorts were more outgoing and competitive and therefore scored more highly on extraversion as a result. This may also be reflected in the substance use behaviour of these cohorts, as preliminary data suggest that competitive gamers consume substances with stimulating effects [95]. This may also be associated with the low scores in agreeableness that were found among the at-risk and high-risk cohorts because low agreeableness is associated with competitive and antagonistic attitudes [96]. How personality factors may influence individuals’ engagement with different videogame genres is beyond the scope of the present study. However, the present study highlights the need to investigate the ambiguity found in relation to extraversion further.

Emotional stability appeared as the lowest scored trait across each of the at-risk and high-risk cohorts. However, it should be noted that the UK cohort appeared to have higher emotional stability within the high-risk profile, although this was not significantly higher than the other two cohorts. Nevertheless, poor emotional stability (i.e., neuroticism) indicates that individuals in the at-risk group may be more emotionally reactive, and therefore find it more difficult to cope with stressful situations [96]. It then follows that those who have poor emotional stability are more likely to develop emotion-focused and dysfunctional coping strategies as they are more emotionally reactive.

### 4.4. Coping

In regard to coping, the findings suggest that coping style appeared to be associated with disordered behaviour, and similar to personality factors, this appeared to be consistent across cohorts. It was found that within both the at-risk and the high-risk profiles, individuals consistently scored higher on dysfunctional coping strategies relative to the sample mean, and higher than both problem-focused and emotion-focused coping. Therefore, the present findings support the broader literature that associates poor coping strategies with potentially addictive behaviours [37]. The high-risk cohort consistently scored higher on dysfunctional coping compared to their at-risk counterparts, which suggests that the coping strategies used are likely well established. This could indicate negative long-term effects on mental well-being [97] and the exacerbation of co-occurring behaviours [41] as demonstrated by consistently high scores across disordered behaviour and substance use. For example, it has been documented that individuals (and particularly gamers) utilise strategies such as escapism to cope with stressors in life [98]. While this is not a maladaptive strategy in and of itself, if relied upon without other strategies, it can exacerbate symptomology [44,97]. Therefore, it is important that when considering disordered behaviours, such as disordered gaming, a focus on how individuals cope with life stressors, and their reason for playing videogames (e.g., stress release, escapism) should be considered irrespective of country of residence or perceived risk level. Indeed, a better understanding of coping strategies and how individuals with different personalities approach life stressors would be beneficial for both at-risk and high-risk groups.

### 4.5. Implications and Future Directions

The present results suggest that individuals within the at-risk profile would likely benefit from psychoeducation as a potential preventative strategy. In addition, psychoeducation which considers coping strategies may be efficacious despite varying cultural backgrounds because each present cohort displayed consistent coping profiles. More specifically, informing at-risk and high-risk individuals about adaptive coping strategies and co-occurrence would be beneficial [99], in conjunction with an understanding of cultural manifestations and shared clinical features (e.g., personality factors [46]) of problematic behaviours and/or substance use could increase resilience. This is especially relevant to the emerging adult sample in the present study. Emerging adulthood (i.e., 18–29 years of age) has been considered to be a distinct transitional development period [100]. Consequently, multiple transitions may occur during this period (e.g., identity, interpersonal relationships, and new adult-like roles) which may create psychological discomfort and can potentially precipitate addictive behaviours. More specifically, addictive patterns may consolidate as maladaptive coping strategies within this transitional period [14]. Therefore, it is important that emerging adults receive psychoeducation and preventative strategies focusing on increasing their resilience.

The present results also suggest that individuals within all high-risk cohorts scored highly across disordered substance use and some addictive behaviours, which may indicate co-occurrence [101]. The present research suggests that when considering potential substance and behavioural addictions, a more nuanced understanding should be considered. A number of studies have considered the effect of co-occurring addictive disorders on treatment efficacy, suggesting that treatment efficacy can be increased when considering not only the primary disorder, but also other secondary problematic behaviour [37,102]. Indeed, research in the field of substance abuse has gained traction when considering an integrated treatment approach [103,104]. The present research suggests that high rates of potentially addictive substances and behaviours are present in large samples of at-risk and high-risk gamers, which suggests that co-occurrence is as well. Therefore, scholars should also investigate the efficacy of integrated, transdiagnostic treatment paradigms when engaging at-risk and high-risk gamers.

### 4.6. Limitations and Strengths

There are some limitations that should be acknowledged. Firstly, the measure used to assess personality factors contained only two questions per personality domain, which resulted in the scale having an adequate omega coefficient. As a result, the conclusions drawn in relation to personality should be interpreted with caution, and future research should consider more psychometrically robust personality measures. Furthermore, the study employed self-report measures for each cohort; therefore, participants may not accurately represent their behaviours related to substance use or problematic behaviours, which may lead to biased reporting. Second, the present study utilised a cross-sectional design, and therefore temporal and casual relationships cannot be argued empirically. Third, it is worth noting that the NZ cohort and the number of non-gamers within the whole sample was quite small, which means that the results may not be generalisable to the wider general NZ population and may not apply to non-gamers. Fourth, the labels for each group (e.g., high risk, low risk) were decided based on a visual inspection of the data, and thus are subjective in nature. Fifth, the present study collected data from participants before and during the COVID-19 pandemic. Stay-at-home mandates and quarantines and emotional distress associated with the pandemic may have led to fluctuation of involvement in gaming. The present data consisted of samples from three nations, and each had different timelines and regulations in managing the spread of the virus during the pandemic period. Therefore, it is difficult to draw any statistically informed conclusions specific to COVID-19-related effects. Lastly, while the present study contained three individualistic countries, generalisation to other individualistic countries (e.g., USA) may be limited, as individuals in these countries may have different risk factors unique to that specific country. However, this is also one of the strengths of the present study. The present study had a diverse sample from three individualistic countries, which had large to medium sample sizes within each cohort. The present study considered a number of different risk factors using an LPA analysis, and also provided evidence on how individuals with these profiles may or may not differ within similar cultural settings. The study also provides valuable insight into the gaming community and further adds to the call for stronger research into treatment paradigms [94].

## 5. Conclusions

The present study sought to identify latent profiles characterised by unique patterns of disordered behaviours, personality factors, co-occurrence, and coping strategies. In order to do this, an LPA was conducted on 1916 participants from three countries, the United Kingdom (*n* = 561), New Zealand (*n* = 170), and Australia (*n* = 1185). While evidence suggests that a minority of gamers are affected by GD, there appears to be an at-risk cohort who may utilise gaming as a maladaptive coping strategy and other accompanying potentially addictive behaviour or substance use may be exacerbated as a result [105], the manifestation of which can be influenced by cultural elements [94]. Therefore, when considering gamers from similar countries, it is important to be cognisant of the variations found in the manifestations of GD and accompanying potentially addictive behaviours. This would allow for more precise identification of at-risk behaviours, which may result in more favourable treatment outcomes for those who are at-risk or high-risk individuals. In addition, the present study demonstrated there are specific groups of emerging adult gamers who are at risk of developing maladaptive coping strategies, and which is exacerbated by the aforementioned factors. Consequently, clinicians and experts in the field should consider the use of psychoeducation and preventative strategies for such individuals because emerging adulthood is a transitional period in which maladaptive coping strategies may be consolidated [14]. Similarly, it is vital that future studies continue to investigate how cultural factors, individual factors, and their interactions may impact gamers across the lifespan given that the present study suggests that factors can vary, even within countries which are more individualistically orientated.

## Figures and Tables

**Table 1:** Demographics and videogame use information.

Sociodemographic Variables		Total (*N* = 1916) (561, 170, 1185) (UK, NZ, AU)
Gender	Prefer not to say/Other	43 (6, 2, 30)
Country	United Kingdom	561
	New Zealand	170
	Australia	1185
Marital status	Same-sex civil partnership/married	235 (3, 27, 205)
	Separated, but still legally in a same-sex civil partnership/married	13 (0, 15, 8)
	Civil partnership has been dissolved/divorced	32 (2, 5, 25)
	Never registered a same-sex civil partnership/married	1302 (459, 125, 718)
	Prefer not to say/other	334 (97, 8, 229)
Qualification	Postgraduate degree (e.g., MA, PhD)	107 (0, 25, 62)
	Degree (e.g., BA, BSc)	884 (506, 44, 334)
	Professional qualification (e.g., teaching, nursing, accountancy)	372 (28, 2, 342)
	Other vocational/work related qualifications	98 (0, 4, 94)
	Foundation degree/Progression diploma/Advanced diploma/Certificate or equivalent	77 (3, 13, 61)
	A levels/AS levels/VCEs/Higher diploma or equivalent	10 (0, 10, 0)
	GCSEs/CSEs/O levels or equivalent	62 (0, 62, 0)
	No qualifications or education	5 (0, 4, 1)
	Prefer not to say/Other	301 (4, 6, 291)
Plays videogames?	Yes	1640 (384, 141, 1115)
	No	276 (177, 29, 70)

**Table 2:** Model fit indices of latent profile analyses for all models compared in UK cohort.

Cohort	Classes	AIC	BIC	Entropy	Prob. min	Prob. max	% min	% max	BLRT *p*-Value
UK	1	28,710.87	28,866.74	1.00	1.00	1.00	1.00	1.00	
UK	2	27,955.39	28,193.52	0.77	0.91	0.95	0.42	0.58	0.01
UK	3	27,257.83	27,578.23	0.85	0.92	1.00	0.05	0.52	0.01
**UK**	**4**	**27,095.99**	**27,498.66**	**0.79**	**0.84**	**1.00**	**0.05**	**0.39**	**0.01**
NZ	1	8737.85	8850.74	1.00	1.00	1.00	1.00	1.00	
NZ	2	8423.71	8596.18	0.94	0.93	0.99	0.18	0.82	0.01
NZ	3	8287.44	8519.49	0.94	0.92	1.00	0.07	0.74	0.01
**NZ**	**4**	**8170.85**	**8462.48**	**0.91**	**0.91**	**1.00**	**0.06**	**0.57**	**0.01**
AU	1	60,585.91	60,768.70	1.00	1.00	1.00	1.00	1.00	
AU	2	58,451.02	58,730.28	0.84	0.93	0.97	0.33	0.67	0.01
AU	3	57,974.18	58,349.91	0.76	0.86	0.91	0.25	0.38	0.01
**AU**	**4**	**56,963.68**	**57,435.89**	**0.81**	**0.85**	**0.96**	**0.10**	**0.35**	**0.01**

The selected model specification bolded. AIC is Akaike’s Information criteria; BIC is Bayesian Information Criteria; BLRT is Bootstrap Likelihood Ration Test.

**Table 3:** Standardised score from the sample mean in the United Kingdom cohort.

Variable	At-Risk	High-Risk	Low-Risk/Extraversion	Low-Risk/Introversion
Videogaming	0.40	0.86	−0.40	−0.28
Shopping	0.47	0.67	−0.42	−0.31
Sexual activity	0.41	0.59	−0.29	−0.35
Social media use	0.66	0.42	−0.41	−0.54
Internet use	0.77	0.83	−0.60	−0.55
Drug use	0.26	0.91	−0.12	−0.38
Alcohol use	0.31	1.00	−0.17	−0.41
Cigarette use	0.08	0.82	−0.07	−0.17
Exercise	0.03	0.41	0.07	−0.19
Gambling	−0.15	1.00	−0.22	−0.24
Extraversion	0.01	0.15	0.51	−0.59
Agreeableness	−0.23	−0.49	0.45	−0.08
Conscientiousness	−0.35	−0.53	0.46	0.07
Emotional stability	−0.39	0.03	0.48	0.02
Openness to experience	0.04	−0.46	0.47	−0.49
Problem-focused coping	0.14	−0.04	0.48	−0.70
Emotion-focused coping	0.26	0.04	0.39	−0.78
Dysfunctional coping	0.61	0.64	−0.39	−0.52

**Table 4:** Standardised score from the sample mean in the New Zealand cohort.

Variable	At-Risk	High-Risk	Low-Risk/Extraversion	Low-Risk/Introversion
Videogaming	0.68	0.51	−0.14	−0.28
Shopping	0.70	0.71	−0.38	−0.31
Sexual activity	0.62	1.00	−0.48	−0.35
Social media use	1.00	0.62	−0.34	−0.54
Internet use	1.00	0.67	−0.55	−0.55
Drug use	−0.23	1.00	−0.33	−0.38
Alcohol use	0.11	0.94	0.15	−0.41
Cigarette use	−0.08	1.00	0.50	−0.17
Exercise	−0.06	0.06	−0.72	−0.19
Gambling	−0.09	1.00	−0.20	−0.24
Extraversion	−0.30	0.31	−0.28	−0.59
Agreeableness	−0.09	−0.45	−0.44	−0.08
Conscientiousness	−0.55	−1.30	−0.21	0.07
Emotional stability	−0.81	−0.71	−0.09	0.02
Openness to experience	−0.12	0.57	−0.66	−0.49
Problem-focused coping	0.03	−0.05	−1.42	−0.70
Emotion-focused coping	0.11	0.46	−1.25	−0.78
Dysfunctional coping	0.93	1.00	−0.80	−0.52

**Table 5:** Standardised score from the sample mean in the Australian cohort.

Variable	At-Risk	High-Risk	Low-Risk/Extraversion	Low-Risk/Introversion
Videogaming	0.85	0.47	−0.39	−0.45
Shopping	0.60	0.34	−0.34	−0.27
Sexual activity	0.46	0.49	−0.12	−0.39
Social media use	0.76	0.43	−0.26	−0.49
Internet use	0.99	0.62	−0.47	−0.54
Drug use	−0.27	1.00	−0.23	−0.33
Alcohol use	0.05	1.00	−0.13	−0.28
Cigarette use	−0.09	0.86	−0.09	−0.10
Exercise	0.11	0.01	0.13	−0.20
Gambling	0.29	0.80	−0.27	−0.23
Extraversion	−0.18	0.17	0.35	−0.21
Agreeableness	−0.35	−0.28	0.29	0.10
Conscientiousness	−0.41	−0.63	0.34	0.20
Emotional stability	−0.49	−0.41	0.25	0.28
Openness to experience	−0.24	0.11	0.42	−0.2
Problem-focused Coping	0.12	0.24	0.71	−0.76
Emotion-focused Coping	0.18	0.33	0.63	−0.77
Dysfunctional coping	0.68	0.96	−0.13	−0.69

**Table 6:** At-risk profile post-hoc games Howell pairwise comparisons.

Variables	Cohort 1	Cohort 2	Estimate	Confidence Low	Confidence High	Adjusted *p*-Value
Drug Use	AU	NZ	−0.002	−0.171	0.167	1.00
**Drug Use**	**AU**	**UK**	**0.538**	**0.350**	**0.727**	**<0.001**
**Drug Use**	**NZ**	**UK**	**0.541**	**0.305**	**0.776**	**0.001**
**Gambling**	**AU**	**NZ**	**−0.390**	**−0.702**	**−0.078**	**0.011**
**Gambling**	**AU**	**UK**	**−0.442**	**−0.625**	**−0.260**	**<0.001**
Gambling	NZ	UK	−0.052	−0.328	0.223	0.889
**Internet Use**	**AU**	**NZ**	**0.410**	**0.0580**	**0.763**	**0.019**
**Internet Use**	**AU**	**UK**	**−0.244**	**−0.411**	**−0.076**	**0.002**
**Internet Use**	**NZ**	**UK**	**−0.655**	**−1.014**	**−0.295**	**<0.001**
Videogaming	AU	NZ	−0.183	−0.745	0.378	0.707
**Videogaming**	**AU**	**UK**	**−0.481**	**−0.697**	**−0.265**	**<0.001**
Videogaming	NZ	UK	−0.298	−0.868	0.272	0.419

Note. With adjusted Tukey *p*-value. Bolded rows indicate significance.

**Table 7:** High-risk profile post-hoc games Howell pairwise comparisons.

Variables	Cohort 1	Cohort 2	Estimate	Confidence Low	Confidence High	Adjusted *p*-Value
**Drug use**	**AU**	**UK**	**−1.672**	**−2.454**	**−0.89**	**<0.001**
Drug use	AU	NZ	0.638	-0.117	1.393	0.101
**Drug use**	**UK**	**NZ**	**2.311**	**1.314**	**3.308**	**<0.001**
**Gambling**	**AU**	**UK**	**3.230**	**2.525**	**3.935**	**<0.001**
Gambling	AU	NZ	0.968	−1.863	3.800	0.626
Gambling	UK	NZ	−2.262	−5.108	0.584	0.124

Note. With adjusted Tukey *p*-value. Bolded rows indicate significance.

## Data Availability

The data presented in the present study are available on request from the corresponding author. The data are not publicly available due to the data being used in a larger doctoral project which is under examination at the time of writing.
